# Computational analysis of the electromechanical consequences of short QT syndrome

**DOI:** 10.3389/fphys.2015.00044

**Published:** 2015-02-11

**Authors:** Christopher L.-H. Huang

**Affiliations:** Physiological Laboratory, University of CambridgeUK

**Keywords:** short QT syndrome, cardiac arrhythmia, sudden cardiac death, potassium channels, computational analysis, electromechanical coupling

Exceptional scientific work inquires into new areas or utilizes techniques circumventing existing limitations to study. Alternatively, it may provide insights extending or complementing existing understanding, or generate hypotheses amenable to or worthy of further testing. Adeniran et al. (2013) complete a sequence of physiological papers fulfilling several of these criteria. These address the familial, relatively recently described, cardiac arrhythmic disease of short QT syndrome (SQTS) (Gussak et al., 2000). SQTSs are electrocardiographically characterized by shortened QT intervals (~ 320 ms) and peaked T-waves despite normal cardiac anatomy (Patel and Pavri, 2009). They are clinically associated with syncope, shortened atrial and ventricular effective refractory periods and increased atrial and, potentially fatal, ventricular, arrhythmia (Giustetto et al., 2006). They complement the commoner long QT syndromes in terms of arrhythmic mechanisms. Both likely, albeit differently, involve altered refractoriness and tissue vulnerability producing re-entrant, arrhythmic, substrate.

Adeniran et al. (2013) complete a series of papers on SQTS1 (Adeniran et al., 2011) and SQTS3 models (Adeniran et al., 2012) resulting from genetic alterations in *KCNH2* and *KCNJ2* and therefore in repolarizing I_Kr_ (Sun et al., 2011) and I_K1_ ionic currents (Deo et al., 2013) respectively. Classical physiological analysis demonstrated that K^+^ channel openers increase transmural repolarization dispersion and shorten ventricular effective refractory period potentially producing arrhythmogenic substrate in left ventricular wedge preparations (Patel and Antzelevitch, 2008). It is important to bear in mind that although useful tools in gaining physiological insights, pharmacological agents can show non-specificities in their actions. However, analyses using genotypically accurate animal SQTS models avoiding such manipulations are lacking. Adeniran et al. (2011, 2012) had introduced a first paradigm shift complementing relatively sparse available *experimental* data through *computational* explorations of the electrophysiological basis for arrhythmia in SQTS. They used Markov and/or Hodgkin-Huxley formulations developed from experimental data obtained from expression systems modeling *N588K-hERG* and *D172N-Kir2.1* mutations to replicate SQT1 and SQT3. This demonstrated increased tissue vulnerability to premature stimuli and increased tendencies to form and maintain re-entrant excitation waves in both idealized two-dimensional and more realistic three-dimensional tissue (Adeniran et al., 2012).

Adeniran et al. (2013) assessed for potential effects of action potential shortening in SQTS upon human ventricular mechanical dynamics. They explored whether mechano-electric feedback involving stretch-activated channels (Taggart, 1996; Hu and Sachs, 1997; Calaghan et al., 2003) could conversely contribute to dissociation between ventricular repolarization and the end of mechanical systole. This would match clinical observations (Schimpf et al., 2008). Stretch-activated channels have been previously implicated in regulation of electrical activity by altered contractility or volume load (Franz, 1996; Lab, 1996). Previous studies modeling arrhythmogenesis in SQTS had not extended to considering mechanical properties. This entailed ambitious computational compilation of physiological, anatomical and biophysical data into a cells-to-systems reconstruction of cardiac electrophysiological activation, contraction and relaxation.

At the cellular level, an established electrophysiological single cell model recapitulated human ventricular myocyte electrical and membrane channel properties as well as transmural action potential timecourse heterogeneities across the ventricular wall (Ten Tusscher et al., 2004; Ten Tusscher and Panfilov, 2006). This yielded cytosolic [Ca^2+^], sarcoplasmic reticular (SR) and cytoplasmic volumes, SR-Ca^2+^ leak and pump currents, Ca^2+^ current from dyadic to bulk cytoplasmic space, and background, plateau and Na^+^/Ca^2+^ exchange membrane Ca^2+^ current. The resulting Ca^2+^-troponin binding coupled this to a myofilament mechanics model chosen for its realistic basis in the cross-bridge cycling model of cardiac muscle contraction (Rice et al., 2008). This latter model has replicated a wide range of experimental data including steady-state force-sarcomere length, force-Ca^2+^ and sarcomere length-Ca^2+^ relations. The simulations compared results from inclusion or exclusion of stretch-activated current, *I*_SAC_.

These cellular level findings were built into predictions for mechanical activity in two and three-dimensional human ventricular tissue models. The modeling of cardiac tissue mechanics permitted a non-linear elasticity (Marsden and Hughes, 1994; Holzapfel, 2000) within an inhomogeneous, anisotropic, incompressible non-linear material (Niederer and Smith, 2008; Pathmanathan and Whiteley, 2009). Including appropriate stress tensors permitted representation of active tension driving longitudinal shortening, wall thickening and rotational twisting of the ventricular wall (Cheng et al., 2008; Coppola and Omens, 2008; Lilli et al., 2013). The geometrical changes were incorporated into the electrophysiological computations (Pathmanathan and Whiteley, 2009). The three-dimensional simulations of human ventricular geometry employed anisotropic fiber orientation data to a 0.2 mm spatial resolution obtained from diffusion-tensor magnetic resonance imaging. This distinguished endocardial, M-cell and epicardial regions.

The action potential shortening associated with SQT did appear to reduce ventricular mechanical function. Furthermore, this was rectified by an inclusion of stretch-activated channels. This prediction justifies future experimental testing as to whether such channels indeed exert functional effects on cardiac electro-mechanical coupling in SQTS1 and SQTS3. The experimental testing might then explore involvements of *I*_SAC_ in normal as well as SQT hearts. In addition, at the theoretical level, the present modeling inevitably entails assumptions of particular implicit physical mechanisms operating within the reconstructions of physiological behavior. It did yield predictions matching previous experimental force-frequency data over frequencies including that at which the simulations were conducted. As outlined in the limitations section of the article, further validation including improved and more physiologically detailed representations of Ca^2+^ dynamics (Iribe et al., 2006; Grandi et al., 2010; O'Hara et al., 2011) as further experimental data becomes available, will help test uniqueness in the model's predictions. Finally, future predictions of mechanical events might fully encompass ventricular pressure, *P*, its derivative, d*P*/d*t*, as well as the timecourse and amplitude of chamber contraction and relaxation.

Nevertheless, the approach adopted by the authors draws attention to the potential value for modeling that integrates cellular electrophysiological, Ca^2+^ homeostatic and biomechanical changes with realistic descriptions of cardiac anatomy and mechanical properties. At the broader level we look forward to applications and developments of such approaches at the whole organ level in the analysis of other ion channel exemplars of abnormal cardiac physiology hitherto mainly studied at the cellular or tissue levels. These might include models for the long QT, Brugada and catecholaminergic polymorphic ventricular tachycardia, syndromes (Thomas et al., 2008; Martin et al., 2011; Zhang et al., 2013).

## Conflict of interest statement

The author declares that the research was conducted in the absence of any commercial or financial relationships that could be construed as a potential conflict of interest.
